# Assisted reproductive technologies in Latin America: the Latin American Registry, 2022

**DOI:** 10.5935/1518-0557.20260017

**Published:** 2026

**Authors:** Fernando Zegers-Hochschild, Javier A. Crosby, Carolina Musri, Fanny Petermann-Rocha, Gustavo Martinez, Hitomi Nakagawa, Carlos Morente, Armando Roque, Ana Palma-Govea, on behalf of the Latin American Network of Assisted Reproduction

**Affiliations:** 1 Program of Ethics and Public Policies in Human Reproduction, Faculty de Medicine, University Diego Portales, Santiago, Chile; 2 Unit of Reproductive Medicine, Clínica Las Condes, Santiago, Chile; 3 SG Fertility Chile, Chile; 4 Medicina Reproductiva Fertilis, San Isidro, Buenos Aires, Argentina. Universidad de Belgrano, Buenos Aires, Argentina; 5 GENESIS, Centro de Assistência em Reprodução Humana, Brasilia, Brazil; 6 Centro Médico PROAR. Rosario, Argentina; 7 Centro Especializado de Atención a la Mujer (CEPAM), Hacienda de las Palmas, Huixquilucan, Estado de México, Mexico; 8 IVI Panamá, Ciudad de Panamá, Panama; 9 Latin American Network of Assisted Reproduction (REDLARA), Montevideo, Uruguay

**Keywords:** ART Registry, reproductive outcome, oocyte donation, PGT, embryo aneuploidy

## Abstract

**Research question:**

What are the trends and impact of ART interventions on the effectiveness and safety of ART performed in Latin America during 2022.

**Design:**

Retrospective collection of cycle-based multinational data obtained from ART procedures performed by 204 accredited institutions in 16 countries.

**Results:**

In total 123,265 initiated cycles resulted in 19,663 deliveries and 22,203 births. ART utilization showed great variability, from 643.3 cycles/million inhabitants in Uruguay to 28.8 in Guatemala. In autologous cycles, the proportion of women ≥40 years represents 35.1% of cycles while women <34 years represents only 18.8%. Nonetheless, the proportion of single embryo transfer (SET) increased from 42.4% in 2021 to 47.3% in 2022. Out of 22,203 babies born, 77.4% were singletons, 21.7% twins and 0.9% triplets or more. ICSI represented 85.4% of fertilization techniques and blastocyst transfer increased from 79.3% in 2021 to 85.3% in 2022. The delivery rate after fresh blastocyst elective SET (32.6%) was significantly higher than after the transfer of one FET from freeze-all cycle (25.2%) (*p*=0.0001). The number of aspirations leading to preimplantation genetic testing has increased 2.6 times in 6 years, and significantly increased delivery rates/transfer (*p*≤0.008) and reduced miscarriage at all ages (*p*≤0.004) in autologous cycles, but not in oocyte donation cycles. Delivery rates after fresh transfer of embryos from vitrified-warmed donated oocytes, generated similar outcome to FET/OD (*p*=0.5621). Perinatal mortality increased from 7.5‰ in singletons to 22.8‰ in twins.

**Conclusion:**

Systematic collection of cycle-based multinational data contributes to cooperative sustained development and helps implement evidence-based reproductive decisions.

## INTRODUCTION

This is the 34^th^ report of the Latin American Registry of Assisted Reproduction (RLA). Publications from 1990 to 2011 can be found at www.redlara.com. Since 2012, reports have been published simultaneously in Reproductive Bio-Medicine Online RBMO and in JBRA Assisted Reproduction, the official journals of the Latin American Network of Assisted Reproduction (REDLARA). The data collection system consists of a cycle-based multinational registry which provides detailed information on utilization, availability, effectiveness, safety, and perinatal outcomes of ART treatments performed in Latin America. This report contains information on ART cycles initiated between January 1^st^ and December 31^st^, 2022, and babies born up to September 2023. When relevant, longitudinal analyses have been used to examine trends over the past decades.

## MATERIALS AND METHODS

Data on ART were collected from 204 centers in 16 countries (Supplementary Table 1), covering fresh autologous cycles of in vitro fertilization (IVF) and intracytoplasmic sperm injection (ICSI); preimplantation genetic testing (PGT); frozen embryo transfer (FET) preceded by both fresh embryo transfer cycles and from freeze-all cycles; oocyte donation (OD), including the transfer of fresh and frozen-thawed embryos resulting from fresh donated oocytes; fertility preservation (FP); and embryo transfer cycles of embryos developed from vitrified-warmed oocytes (VWO), both autologous and heterologous (OD).

An independent body within REDLARA has accredited all institutions reporting to RLA. The forms used for this process can be obtained at www.redlara.com. Participating centers agree to have their data published by RLA. Therefore, no specific consent forms were requested for the scientific disclosure of data. The method of data collection in 2022 resembles previous years (Zegers-Hochschild *et al*., 2020), making results comparable. The definitions used are those published in the International Glossary on Infertility and Fertility Care (Zegers-Hochschild *et al*., 2017). Cases of total embryo freezing have been excluded when calculating clinical pregnancy or delivery rates per oocyte retrieval. Furthermore, clinical pregnancies with lost to follow-up (LFU) were also excluded from the calculation of delivery rates.

The cumulative delivery rate was calculated from aspirations and their related fresh and frozen transfer cycles taking place between January and December 2022. Data included 204 institutions in 16 countries. Results were expressed as: a) cumulative delivery rate starting with all fresh transfers, irrespective of whether there were supernumerary frozen embryos; and b) cumulative deliveries including only women having surplus frozen embryos apart from their fresh transfers.

### Statistical analyses

To test the association of age, number of embryos transferred, and stage of embryo development at transfer on the delivery rate per embryo transfer (DR/ET), Poisson regression models with robust standard errors were used to analyse cross-sectional associations. The results are reported as prevalence ratios (PR) with their 95% confidence intervals (CIs). Poisson regression models with robust standard errors were used because they provide PR estimates which are relatively easy to interpret instead of odds ratios (Grant, 2014). Robust standard errors were used to correct underinflation when applying the Poisson model for binary outcomes. When variables were not stratified by age, analyses were adjusted for it. *p*<0.05 was considered statistically significant. Stata 18 statistical software (Stata Corp LP) was used to perform all analyses.

## RESULTS

A total of 204 centers in 16 countries reported 123,265 initiated cycles (3.2% fewer than in 2021), which resulted in 19,663 deliveries, 22,203 births and adding the estimated births in nonreporting institutions from reporting countries, we can estimate a total of 26,693 births in the region.

Brazil remains the largest contributor with 40.6% of all initiated cycles, followed by Mexico and Argentina with 17.1% and 16.2% cycles, respectively.

Fresh IVF and ICSI cycles represented 27.8% of initiated cycles, followed by autologous FET with 22.0% and aspirations leading to PGT and transfer cycles after PGT represented 20.9% of all cycles. Oocyte donation and the transfer of vitrified warmed oocytes (VWO) were 13.7% and 4.9% respectively (Table 1). Furthermore, a detailed breakdown of events that start with initiated ART cycles up to when embryos are transferred can be seen in Figure 1. From 34,213 initiated fresh IVF and ICSI cycles, excluding PGT, embryo transfer took place in only 11,495 cycles (33.6%). Part of this reduction resulted from 12,697 freeze all cycles, representing 38.7% of aspirations. A similar exercise can be done with the rest of treatment categories represented in Figure 1.

**Table 1:** Assisted Reproduction Techniques reported in Latin America, 2022.

Country	Centers	FP	Autologous Fresh IVF/ICSI^*^	FET (own)^*^	OD^*^ Fresh/FET	Fresh VWO^*^	FET VWO^*^	PGT^**^	Total	Deliveries registered by RLA	Total number of births registerred by RLA	Estimated total number of lifebirths from ART	Estimated proportion lifebirths from ART/ total births in the country
**Argentina**	17	1778	6859	4780	3992	463	567	1499	**19,938**	2823	2971	3129	0.51
**Bolivia**	4	12	294	53	270	25	0	24	**678**	152	184	326	0.13
**Brazil**	68	6629	13,236	12,997	3349	2178	569	11,142	**50,100**	7267	8226	9851	0.39
**Chile**	11	998	2206	1599	726	350	178	3078	**9135**	1394	1450	1714	0.91
**Colombia**	16	390	1531	992	905	216	55	633	**4722**	786	892	1039	0.16
**Costa Rica**	1	7	97	46	11	3	0	2	**166**	17	18	38	0.07
**Ecuador**	8	83	558	210	326	38	0	151	**1366**	282	317	395	0.16
**El Salvador**	1	24	33	6	19	2	0	12	**96**	10	13	34	0.04
**Guatemala**	2	13	115	73	101	6	0	74	**382**	88	111	145	0.04
**Mexico**	47	1233	6440	4360	4510	409	58	4095	**21,105**	4115	5037	5918	0.36
**Panama**	4	114	266	280	161	38	0	348	**1207**	188	204	372	0.58
**Paraguay**	1	36	167	121	74	2	0	67	**467**	75	82	167	0.17
**Peru**	17	1795	1623	1157	2008	579	188	4414	**11,764**	2010	2205	2812	0.67
**Rep. Dom.**	2	20	161	51	89	8	1	26	**356**	87	96	202	0.13
**Uruguay**	2	94w	556	403	341	50	3	203	**1650**	346	373	497	1.03
**Venezuela**	3	1	71	25	30	3	0	3	**133**	23	24	54	0.01
**Total %**	**204**	**13,227**	**34,213**	**27,153**	**16,912**	**4370**	**1619**	**25,771**	**123,265**	**19,663**	**22,203**	**26,693**	**0.33**
		**10.7%**	**27.8%**	**22.0%**	**13.7%**	**3.5%**	**1.3%**	**20.9%**	**100%**				

FP, fertility preservation; FRESH, initiated fresh autologous IVF/ICSI cycles; FET, initiated autologous frozen embryo transfer; OD, initiated oocyte donation with fresh or frozen/thawed embryos; VWO, initiated autologous and donated vitrified/warmed oocytes cycles.

* Without PGT cycles;

** PGT include aspiration cycles leading to PGT and FET cycles after PGT from autologous IVF/ICSI, OD and VWO.

**Figure 1:**
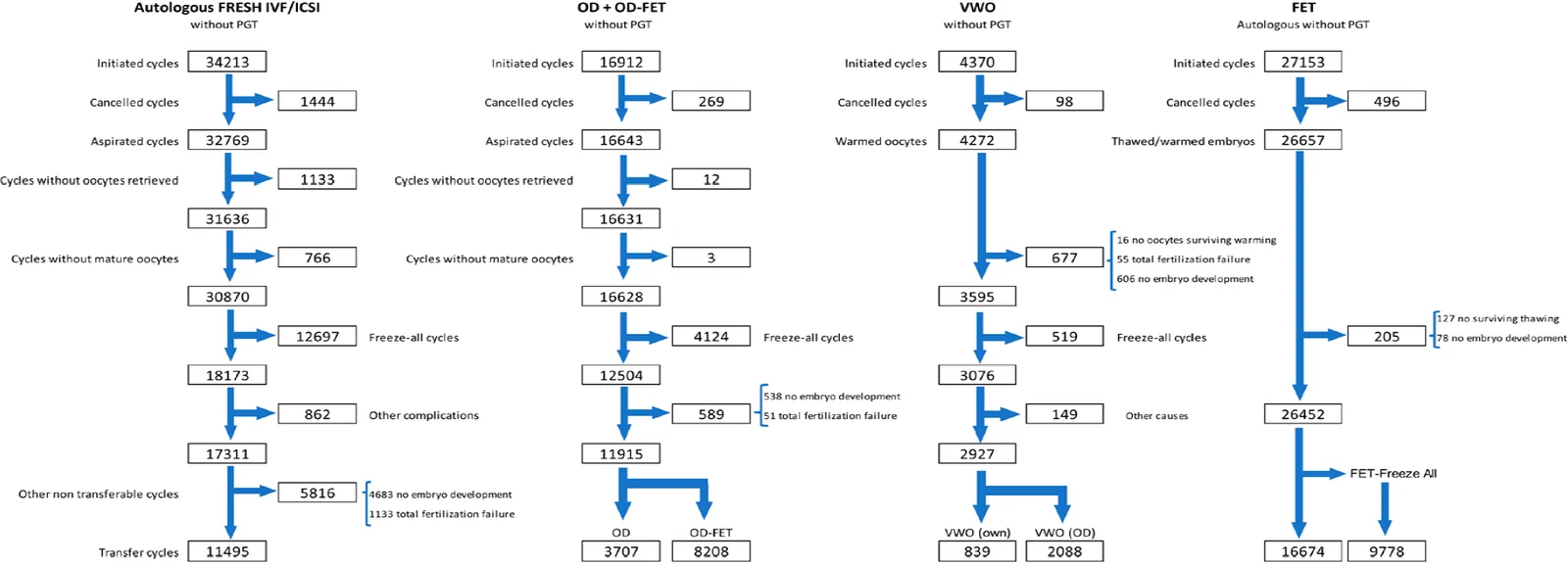
Events that affect the outcome of Fresh in vitro fertilization and intracytoplasmic sperm injection (IVF/ICSI), fresh and frozen oocyte donation (OD), vitrified warmed oocyte (VWO) and autologous frozen embryo transfer (FET) in Latin American ART Registry, 2022.

### Utilization of assisted reproductive technology in Latin America

Utilization of ART is expressed as the total number of ART cycles performed per million inhabitants. As in previous years, calculations of ART utilization, included reported cycles plus the best estimate of non-reported cycles from countries having at least one center reporting to RLA. The magnitude of the estimates was expressed as degrees of confidence according to Dyer *et al*. (2019) and later applied by Zegers-Hochschild *et al*. (2021). As seen in Figure 2, RLA collects more than 80% of cycles performed in major contributing countries (Brazil, Mexico, Argentina, Peru, Chile, Colombia).

**Figure 2:**
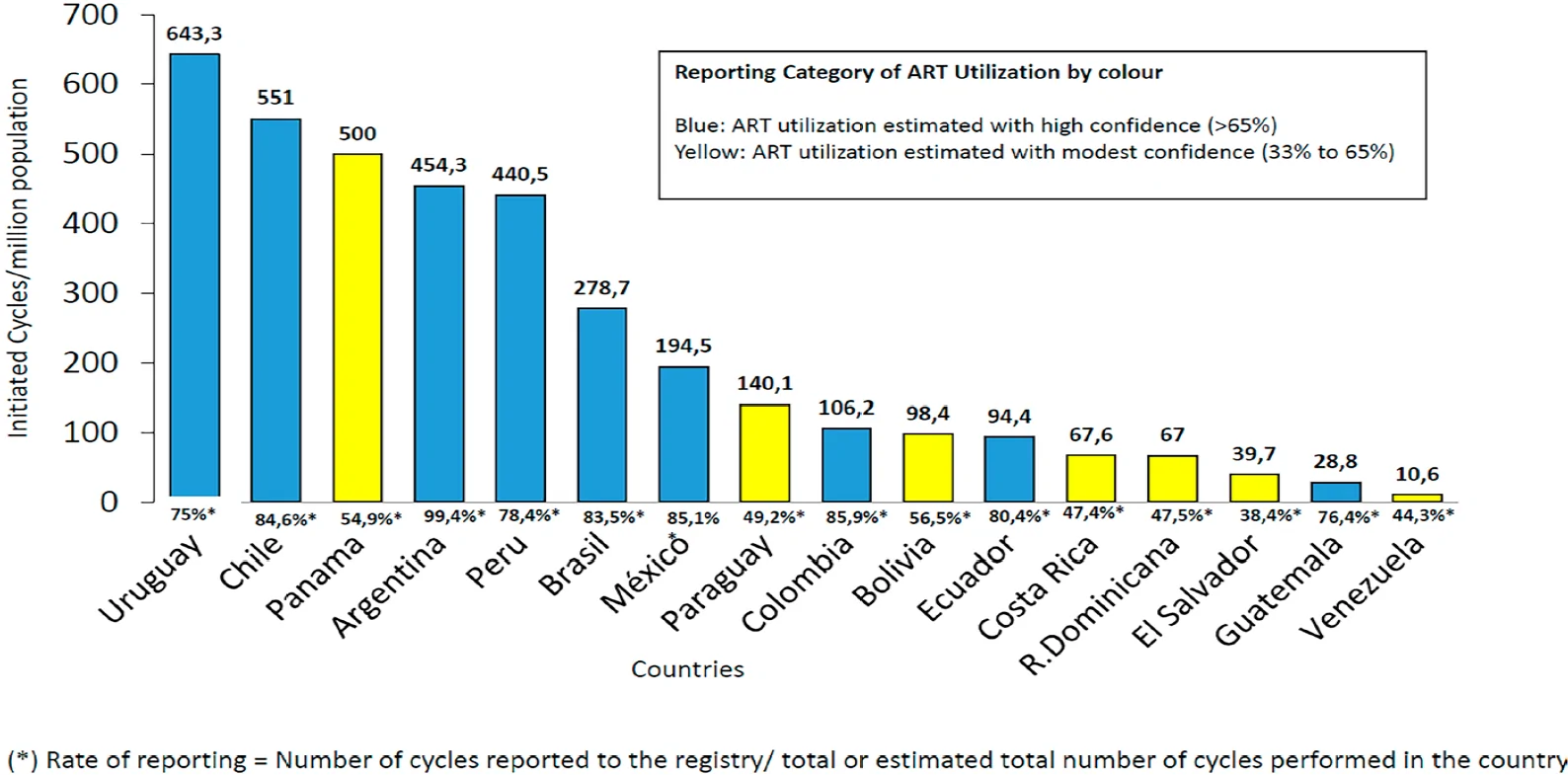
Utilization of assisted reproductive technology. Estimated number of initiated cycles per million inhabitants by country in Latin American ART Registry, 2022.

### Age of women and number of embryos transferred

The age distribution of women treated with autologous reproduction has remained unchanged compared with our previous report. 76% of women were ≥35 years and 35.7% were ≥40 years. Despite this reproductively aging population, the mean number of embryos transferred continue to drop from 1.7 to 1.6 and the proportion of SET increased from 42.4% in 2021 to 47.3% in 2022 (data not shown). Trends in the number of embryos transferred between 1990 and 2021 can be found in Zegers-Hochschild *et al*. (2025).

### Outcome of autologous fresh IVF and ICSI cycles according to the age of women and number of embryos transferred

In 2022, there were 34,213 fresh initiated IVF/ICSI cycles, of which 85.4% were ICSI. After discarding freeze-all cycles and other conditions resulting in no embryos for transfer, the number of transfer cycles dropped to 11,495 (Figure 1). The clinical pregnancy rates (CPR) and delivery rates (DR) per oocyte retrieval and embryo transfer, stratified by the age of women is shown in Table 2. The very low CPR (14.8%) and DR (11%) per oocyte retrieval is in part explained by 2761 aspirations with either no oocytes retrieved, or no mature oocytes apart from other complications (Figure 1). Both CPR and DR were significantly higher when transferring two compared with one embryo (both *p*<0.0001). Furthermore, transferring three embryos only impacted the rate of multiple births, which increased from 1.9% after SET to 20.1% and 29.6% after DET and TET, respectively (Supplementary Figure 1).

**Table 2:** Clinical pregnancy rate and delivery rate in fresh autologous IVF and ICSI cycles stratified according to the age of women in 2022.

Age of women	Oocyte retrievals	Oocyte retrievals^*^	Oocyte retrievals^**^	Clinical Pregnancies (CPR/OR^*^)	Deliveries (DR/OR^**^)	PR (95% CI); *p*-value	Embryo transfers^***^	Deliveries (DR/ET^***^)	PR (95% CI); *p*-value
≤30	3698	1504	1457	437 (29.1)	338 (23.2)	1.00 (Ref.)	955	338 (35.4)	1.00 (Ref.)
31-33	5053	2245	2208	644 (28.7)	521 (23.6)	1.02 (0.90; 1.15); 0.781	1472	521 (35.4)	1.00 (0.90; 1.12); 0.999
34-36	9445	4494	4426	1044 (23.2)	807 (18.2)	0.79 (0.70; 0.88); <0.001	2509	807 (32.2)	0.91 (0.82; 1.01); 0.068
37-39	11,963	6572	6505	1052 (16.0)	761 (11.7)	0.50 (0.45; 0.57); <0.001	3061	761 (24.9)	0.70 (0.63; 0.78); <0.001
40-42	11,255	7553	7525	577 (7.6)	360 (4.8)	0.21 (0.18; 0.24); <0.001	2459	360 (14.6)	0.41 (0.36; 0.47); <0.001
≥43	5083	3985	3973	136 (3.4)	75 (1.9)	0.08 (0.06; 0.10); <0.001	871	75 (8.6)	0.24 (0.19; 0.31); <0.001
**Total**	**46,497**	**26,353**	**26,094**	**3890 (14.8)**	**2862 (11.0)**	-	**11,327**	**2,862 (25.3)**	-

After comparing, DR shows no difference between IVF/ICSI we group them together to analyze only the effect of age.

* Oocyte retrievals (OR) excludes cases of total embryo freezing.

** OR excluding cases of total freezing and pregnancies with loss to follow-up (LFU).

*** Embryo transfers (ET) excluding pregnancies with LFU.

CPR/OR^*^: clinical pregnancy rate per oocyte retrieval; DR/OR^**^: delivery rate per oocyte retrieval excluding cases of total freezing and LFU; DR/ET^***^: delivery rate per transfer excluding LFU

### Outcome of autologous IVF and ICSI after elective and non-elective SET and DET

There were 5482 fresh SET and 5423 fresh DET cycles. Each were further stratified into eSET (41.8%) and eDET (42.3%). As seen in Table 3, which includes the transfer of both cleaving embryos and blastocysts, clinical pregnancy rates and delivery rates were significantly greater after eSET than oSET (40.0% and 30.6%, compared with 19.9% and 14.2%, respectively) (PR clinical pregnancy: 1.80 [95% CI: 1.64; 1.96]; <0.001; PR delivery rate: 1.84 [95% CI: 1.65; 2.06]; <0.001). A similar and significant relationship was also established when comparing eDET and oDET (PR clinical pregnancy: 1.47 [95% CI: 1.38; 1.58]; <0.001; PR delivery rate: 1.52 [95% CI: 1.40; 1.65]; <0.001). The impact of eSET over oSET at all ages can be seen in Figure 3.

**Table 3:** Clinical pregnancy rate, delivery rate and gestational order in elective and non-elective SET and DET in fresh autologous IVF/ICSI in 2022.

Type of transfer	Embryo transfers		Clinical pregnancies		Embryo transfers^*^	Deliveries							
	Number	%	Number	%	Number	Number of deliveries	Delivery rate per embryo transfer^*^ (%)	Singleton (n)	Singleton (%)	Twin (n)	Twin (%)	≥Triplets (n)	≥Triplets (%)
oSET	3193	58.2	634	19.9	3147	447	14.2	438	98.0	9	2.0	0	0
eSET	2289	41.8	915	40.0	2241	686	30.6	674	98.3	12	1.8	0	0
oDET	3129	57.7	979	31.3	3046	708	23.2	587	82.9	120	16.9	1	0.1
eDET	2294	42.3	1120	48.8	2228	852	38.2	660	77.5	186	21.8	6	0.7

* Excluding cases of pregnancies with loss to follow-up (LFU).

eSET, elective single-embryo transfers; oSET, transfer of only one embryo because there are no more embryos available for transfer; eDET, elective double-embryo transfers; oDET, the transfer of only two embryos because there are no more embryos available for transfer.

**Figure 3:**
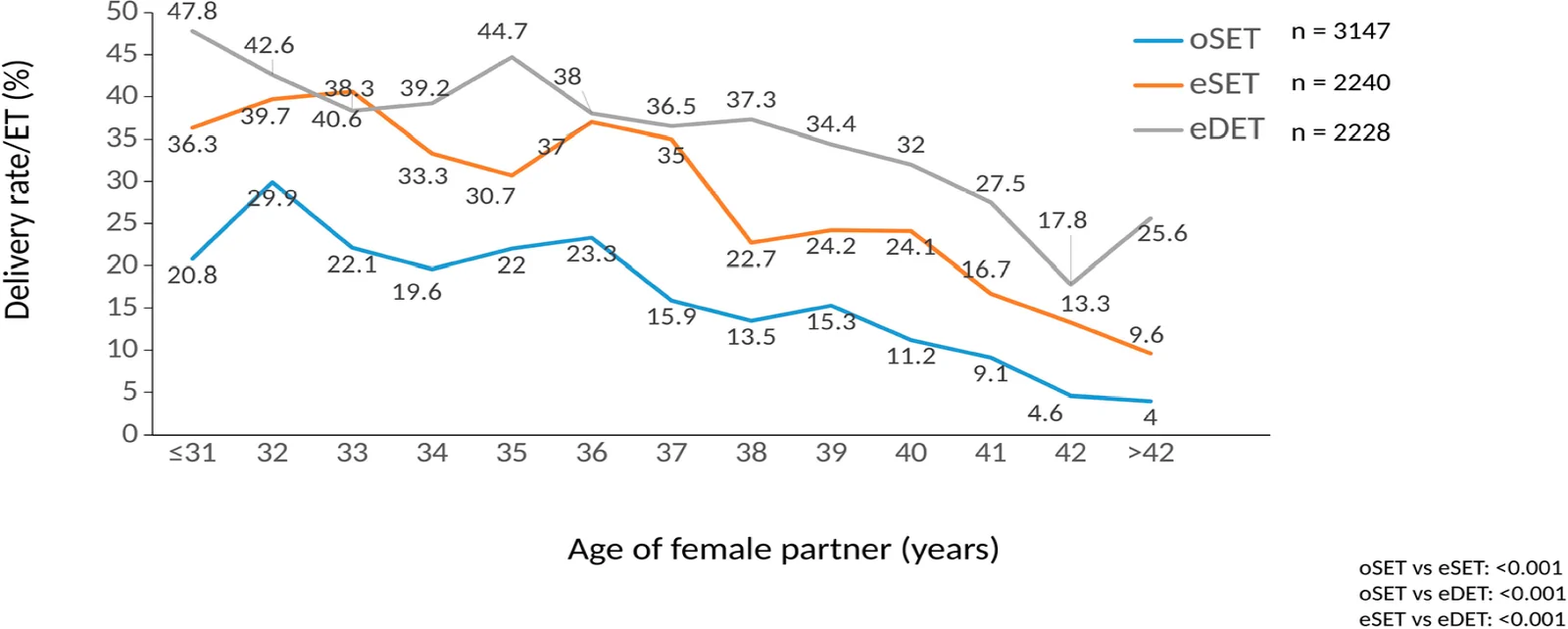
Delivery rate per embryo transfer (DR/ET) in autologous fresh IVF and ICSI cycles according to the age of the female partner and the number of embryos transferred in Latin American ART Registry, 2022. eSET: elective single-embryo transfers; oSET: transfer of only one embryo with no more embryos available for transfer; eDET: elective double-embryo transfers.

### Influence of blastocyst over cleaving embryo transfer

The proportion of blastocyst transfers over cleaving embryos represents one of the major changes impacting reproductive outcome. Today, blastocyst transfer represents 85.3% (54,050/63,350) of all transfers, increasing again compared to 2021 (79.3%). As seen in Supplementary Figure 2, blastulation rate, or the capacity of zygotes to reach blastocyst stage (number of blastocysts transferred + blastocysts vitrified, divided by the number of zygotes generated), has improved in the last 8 years, reaching 46.7% in women ≤34 years, 43.3% in women 35-39 years and 33.9% in women ≥40 years. The number of blastocysts generated reflects reproductive efficiency and has a direct impact on the chances of birth after a fresh transfer. As seen in Figure 4, which includes 6789 blastocyst transfers in 2022, the delivery rate after a fresh transfer is significantly higher in women generating one, two or three extra blastocysts, compared with women having no blastocyst left after their fresh transfer (*p*=0.0001). The generation of ≥ 2 blastocysts increases the chances of birth in the fresh transfer, and also increases the cumulative chances of birth (data shown under cumulative delivery rate).

**Figure 4:**
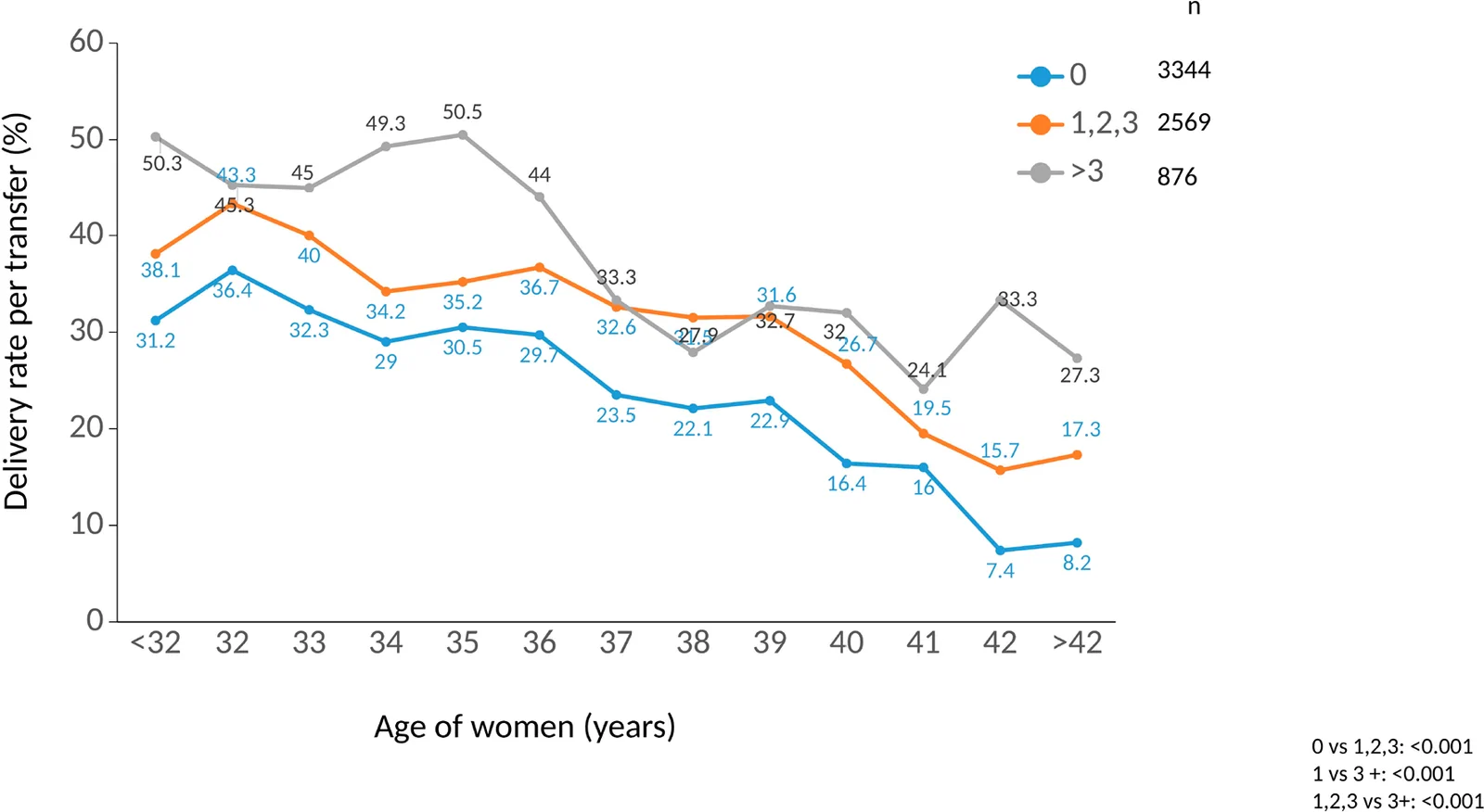
Delivery rate in Fresh transfers of the Latin American ART Registry in 2022 according to the age of women and the number of extra blastocysts vitrified from the same cohort. 0: no other blastocyst vitrified; 1,2,3: one to three extra blastocyst vitrified; 3+: more than 3 extra blastocysts vitrified.

### Frozen embryo transfer cycles

Out of 63,350 embryo transfer cycles, 41,470 were FET cycles of which 33,262 were autologous and 8208 in OD cycles. The proportion of autologous FET over autologous Fresh IVF/ICSI transfers including PGT cases continues to increase over previous years reaching 74.2% (33,262/44,848) of transfers in 2022 (72.5% in 2021) (Zegers-Hochschild *et al*., 2025). In autologous cycles FET has increased from 9.1% in 1996 to 74.2% in 2022. The increasing use of this technology has contributed to a decrease in the number of embryos transferred, from a mean of 3.2 to 1.6 in the last 20 years.

Out of 16,674 autologous FET cycles, excluding PGT and freeze-all cycles (Table 4), the overall CPR, DR and multiple births per transfer were 40.3%, 30.1% and 10.9%, respectively. This better outcome in FET over fresh transfer: 33.6%, 25.3% and 13.4% (values extracted from Supplementary Figure 1) are observed with one and two embryos transferred (SET: *p*<0.0001; DET: *p*<0.0001; respectively). The better outcome of FET over fresh transfers is probably multifactorial, including better endometrial receptivity but most of all, a higher proportion of blastocyst transfer in FET (87.8%) compared with fresh (57.8%) (data not shown).

**Table 4:** Clinical pregnancy rate, delivery rate and gestational order according to the number of embryos transferred in Autologous FET cycles in 2022

Number of embryos transferred	Embryo transfers^*^		Clinical pregnancies		Embryo transfers^**^	Deliveries							
	Number	%	Number	CPR(%)	Number	Number of deliveries	Delivery rate per embryo transfer^**^(%)	Singleton (n)	Singleton (%)	Twin (n)	Twin (%)	≥Triplets (n)	≥Triplets (%)
1	10,306	61.8	3908	37.9	9954	2829	28.4	2778	98.2	50	1.8	1	0.04
2	5937	35.6	2630	44.3	5713	1878	32.9	1422	75.7	450	24.0	6	0.3
≥3	431	2.6	186	43.2	418	137	32.8	117	85.4	18	13.1	2	1.5
Total	16,674	100	6724	40.3	16,085	4844	30.1	4317	89.1	518	10.7	9	0.2

* Excludes vitrified-warmed oocyte, PGT and Freeze all cycles

** Excluding cases of pregnancies with loss of follow-up (LFU).

### Freeze-all cycles

During 2022 there were 12,697 follicular aspirations leading to freeze-all autologous cycles (37.1%) (Figure 1), and 9778 autologous freeze-all transfer cycles resulting from procedures performed in 2022 and in previous years. Freeze-all autologous transfers generated 4110 clinical pregnancies and 2937 deliveries with an overall clinical pregnancy and delivery rate per transfer of 42.0 and 31.2% (Table 5). Clinical pregnancy rate but not delivery rate was significantly higher in freeze all compared with FET resulting from a previously failed fresh transfer (40.3% and 30.1%, respectively (Table 4; CPR *p*=0.0066; DR: *p*=0.0655). Nonetheless, when freeze-all cycles were compared with the elective fresh transfers of only blastocysts (Supplementary table 2), the DR after fresh eSET blastocyst (32.6%) was significantly greater than after the transfer of one embryo after a freeze-all cycles 25.2% (*p*<0.0001; Table 5).

**Table 5:** Clinical pregnancy rate, delivery rate and gestational order according to the number of embryos transferred after Autologous Freeze all cycles, 2022

Number of embryos transferred	Embryo transfers^*^		Clinical pregnancies		Embryo transfers^**^	Deliveries							
	Number	%	Number	CPR(%)	Number	Number of deliveries	Delivery rate per embryo transfer^**^(%)	Singleton (n)	Singleton (%)	Twin (n)	Twin (%)	≥Triplets (n)	≥Triplets (%)
1	4581	46.9	1590	34.7	4436	1118	25.2	1099	98.3	19	1.7	0	0.0
2	4832	49.4	2345	48.5	4640	1698	36.6	1225	72.1	465	27.4	8	0.5
≥3	365	3.7	175	47.9	352	121	34.4	87	71.9	30	24.8	4	3.3
Total	9778	100	4110	42.0	9428	2937	31.2	2411	82.1	514	17.5	12	0.4

* Excludes cases with PGT.

** Excluding cases of pregnancies with loss of follow-up (LFU).

### Endometrial preparation for frozen embryo transfer

Endometrial preparation for embryo transfer was compared between cycles with hormonal replacement versus ultrasound monitoring of a natural cycle. Out of 26,452 autologous FET transfers excluding PGT (Figure 1), 3747 cases (14.2%) were transferred in a monitored natural cycle and 22,705 (85.8%) after endometrial preparation with oral Estradiol and vaginal Progesterone. The mean age of women was 37.0 years in both groups, and the mean number of embryos transferred was 1.49 (SD 0.540) in monitored natural cycle and 1.46 (SD 0.565) in hormonal replacement cycle (*p*=0.0025). The clinical pregnancy rate and delivery rates were 40.8% (1530/3747) and 31.9% (1172/3676) in monitored natural cycles and 41.0% (9304/22,705) and 30.3% (6609/21,837) in hormonal replacement cycles. No differences were found in CPR (*p*=0.8176), but the DR was in the border of significance, favoring the natural cycle (*p*=0.0513). This difference needs to be tested in a properly randomized controlled trial.

### Influence of preimplantation genetic testing (PGT) on ART outcome

In the last 6 years, the proportion of autologous aspirations with at least one mature oocyte leading to PGT increased from 11.5% in 2017 to 30.8% (13,728/44,597) in 2022 (Supplementary Figure 3). When stratified by age, the percentage of aspirations associated with PGT was 19.2% in women ≤34; 31% in women 35 to 39 years and 39% in women ≥40 years. Out of 17,017 procedures were PGT was perfomed, 13,728 were autologous fresh, 1759 were OD and 1530 resulted from VWO (Supplementary Figure 4). Furthermore, in autologous PGT transfers, 21.6% were performed in women ≤34 years, 44.1% in women 35-39 years and 34.4% in women ≥40 years (Table 6).

**Table 6:** Effect of PGT on the delivery rate and miscarriage rate according to age of women in autologous FET and OD FET (2022)

	Age of women	FET with PGT	FET without PGT	PR (95% CI); *p*-value
Delivery^*^	Oocyte Donors	38.0% (823/2167)	35.7% (3287/9204)	0.94 (0.89; 1.00); 0.060^¥¥^
	Autologous <35	40.2% (536/1333)	36.6% (2845/7770)	0.91 (0.84; 0.98); 0.008^¥¥^
	Autologous 35 - 39	39.6% (1079/2724)	30.6% (3255/10,654)	0.77 (0.73; 0.81); <0.001^¥¥^
	Autologous >39	38.0% (807/2125)	24.1% (1779/7393)	0.63 (0.59; 0.68); <0.001^¥¥^
Miscarriage^*^	Oocyte Donors	15.3% (160/1046)	15.8% (680/4310)	1.02 (0.87; 1.20); 0.773^¥^
	Autologous <35	12.1% (84/695)	16.6% (634/3817)	1.36 (1.10; 1.69); 0.004^¥^
	Autologous 35 - 39	12.3% (175/1419)	19.1% (871/4571)	1.54 (1.33; 1.80); <0.001^¥^
	Autologous >39	13.2% (137/1038)	21.4% (551/2572)	1.62 (1.37; 1.93); <0.001^¥^

FET, frozen embryo transfer; OD FET = oocyte donation frozen embryo transfer; PGT, preimplantation genetic testing; PR, prevalence ratio.

All analyses were adjusted for women’s age.

(^*^) For miscarriage the denominator are clinical pregnancies, for deliveries, the denominator are embryo transfers without LFU.

¥ Likelihood of having a miscarriage. The reference group is “with PGT”.

¥¥ Likelihood of delivery. The reference group is “with PGT”.

The effect of PGT on the delivery rate and miscarriage rate can be seen in Table 6. Excluding clinical pregnancies with LFU, there were 8349 FET/PGT cycles, of which 6182 transfers were from autologous cycles (74.0%) and 2167 (26.0%) from OD. Delivery rate per embryo transfer was significantly greater with PGT in all autologous age groups (*p*=0.008 for <35 years, *p*<0.001 for 35-39 years and >39 years age groups); however, it did not differ significantly for oocyte donation cycles (*p*=0.060). Furthermore, with PGT, there were no differences in DR/ET in autologous young and reproductively older women (< 35 *versus* ≥35 years, *p*=0.3892). The rate of miscarriage was significantly lower in autologous PGT cycles in all age groups (*p*=0.004 for <35 years, *p*<0.001 for 35-39 years and >39 years age groups); however, for OD cycles no difference was found (*p*=0.773). Therefore, even when the denominator is embryo transfer, there is no clear benefit using PGT-A in OD cycles. In spite of this, 19% of FET cycles using donated oocytes (OD) cycles included PGT.

### Frequency of aneuploidy in human embryos

In 2022, a total of 43,385 blastocysts were examined for either aneuploidy or single gene defects. The technique most frequently used in Latin America is Next Generation Sequencing (NGS). The rate of aneuploidy was 48% in women<30 years; 50.9% in women 30 - 34 years; 61.2% in women 35 - 39, reaching 75.5% in women ≥40 (data not shown here). These values are very similar to our previous report of 170,242 embryos examined between 2017-2021 (Zegers-Hochschild *et al*., 2025).

### Outcome of oocyte donation cycles

As seen in Table 1, there were 16,912 initiated cycles, representing 13.7% of all cycles performed in the region. After discarding cancellations, freeze-all cycles, PGT and other factors, there were 11,915 embryo transfers of which 3707 (31.1%) were fresh transfers while 8208 (68.9%) were frozen/thawed transfers (Figure 1).

In contrast with autologous reproduction, both delivery rates and miscarriage rates were practically unaffected by the age of oocyte recipients with a mean age of 41.9 (SD 5.67) years (Figure 5 and Table 6). Clinical pregnancy rate, delivery rate and multiple birth rates were examined in 3707 OD fresh transfer cycles, 8208 OD FET cycles and in 2077 OD fresh transfers from VWO (Supplementary Tables 3 and 4 and 5, respectively). When comparing these three modalities, there was a higher delivery rate after fresh embryo transfer (39.3%) compared with FET/OD (36.5%; *p*=0.0094) but not with fresh transfers of VWO/OD (37.2%; *p*=0.1831). Delivery rates after fresh transfer of embryos from vitrified-warmed donated oocytes (VWO/OD: 37.2%), generated similar outcome (*p*=0.5621) to frozen embryo transfer of oocyte donation cycles (FET/OD: 36.5%). However, delivery rate after a frozen thawed transfer, resulting from VWO (two cryopreservation processes, the oocyte and the resulting embryo) resulted in lower clinical pregnancy and delivery rates than the fresh transfer of VWO (30.3% *versus* 37.2%, *p*=0.0001).

**Figure 5:**
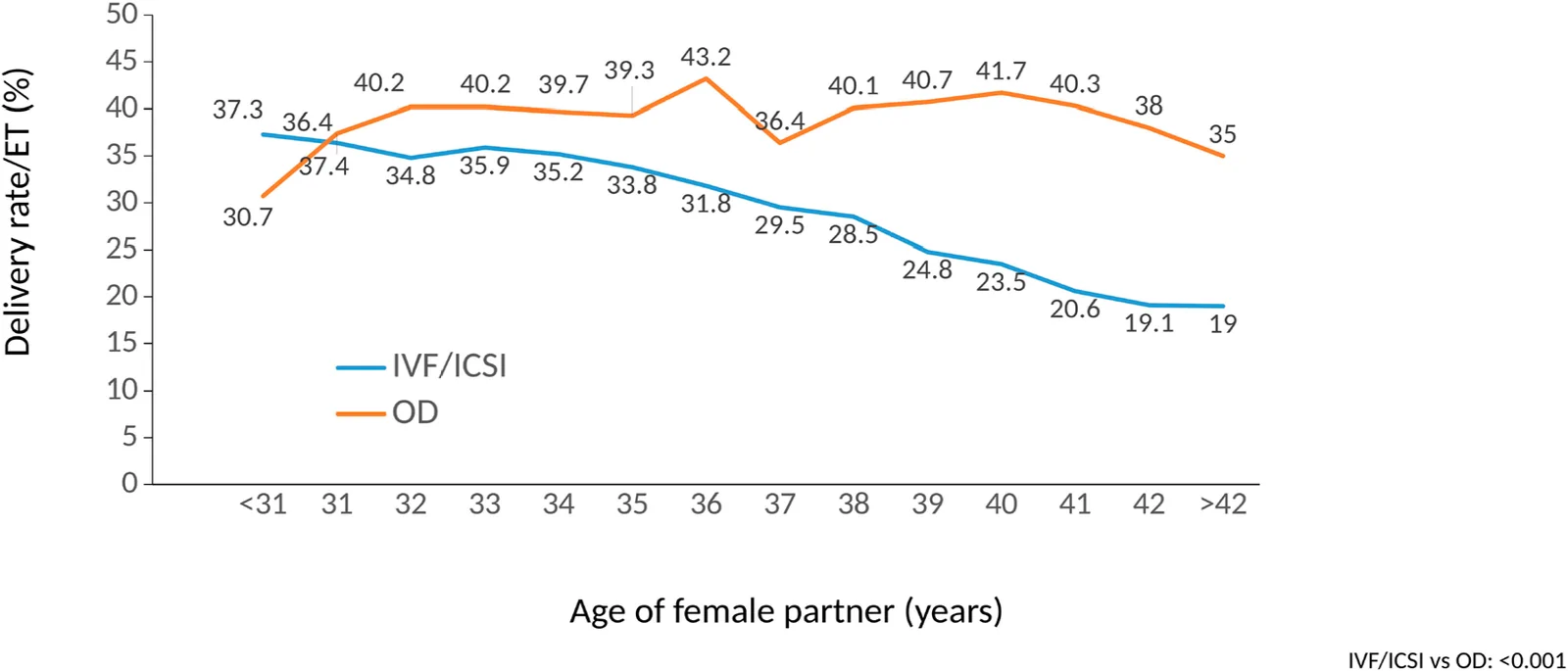
Delivery rate per embryo transfer (DR/ET) in fresh and frozen autologous IVF and ICSI and fresh and frozen oocyte donation (OD) cycles, excluding PGT cycles, according to the age of the female partner in Latin American ART Registry, 2022.

### Cumulative delivery rate

Cumulative delivery rates were calculated in a cohort of 11,327 aspiration cycles irrespective of whether women had surplus frozen embryos for delayed transfer; and in a subgroup of 4584 aspirations where all women had at least one extra embryo frozen for future transfer; irrespective of whether they were used during 2022. For the calculation of cumulative deliveries, this group is the one that better reflects what cumulative chances are, since women that do not have frozen embryos do not have a cumulative chance of birth; their only chance is after the fresh transfer. As seen in Figure 6 (a) and (b), both the fresh and cumulative delivery rates are higher at all ages, apart from age 41 years, in women having frozen embryos for future transfer, which by that mere fact, represent a more fertile population compared to women of the same age but generating only embryos for fresh transfer.

**Figure 6:**
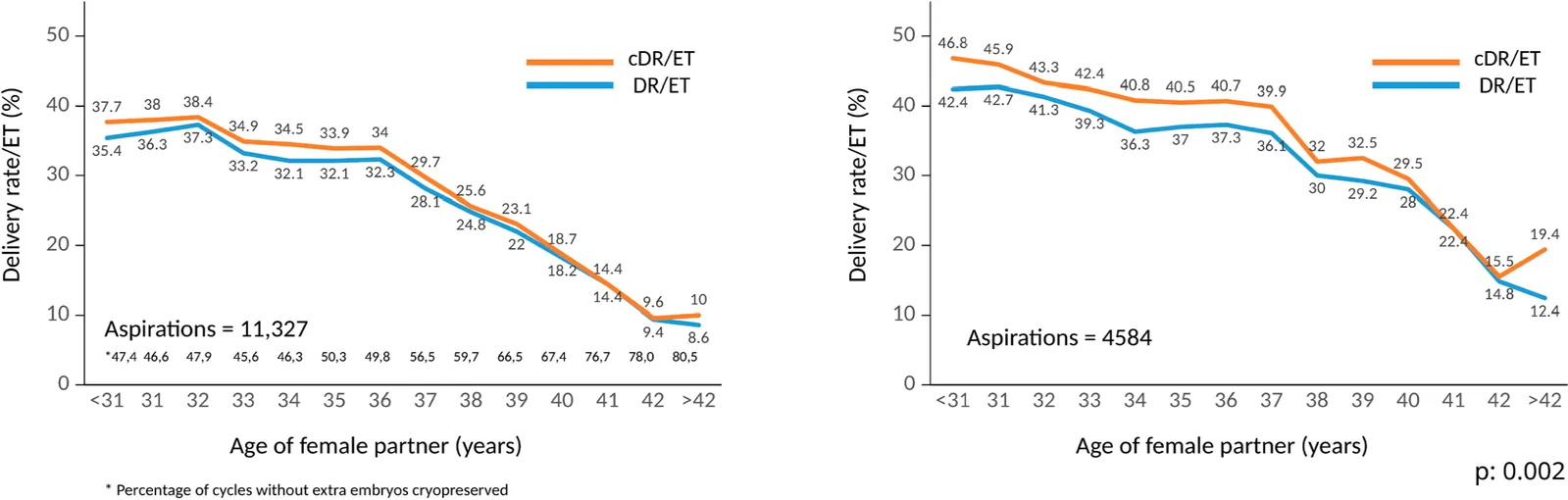
Cumulative delivery rate per embryo transfer (cDR/ET) in IVF and ICSI cycles according to the age of the female partner in Latin American ART Registry, 2022. Left panel: all aspirations irrespective of whether there were frozen embryos for further transfer; right panel: only aspirations with surplus frozen embryos after fresh transfer.

### Perinatal outcome and preterm birth

Perinatal mortality (PNM) and preterm births (Figure 7) were calculated in 22,203 births, of which, 17,189 (77.4%) were singletons, 4820 (21.7%) twins and 194 (0.9%) triplets or more. Consistent with previous years, PNM increased from 7.5‰ in singletons to 22.8‰ in twins and 124.4‰ in triplets and more. On the other hand, preterm birth increased from 15.4% in singletons to 64.4% in twins and 93% in triplets. Also, extreme preterm births (≤32 weeks gestation) increased from 2.7% in singletons to 11.5% and 52.7% in twins and triplets, respectively. In this way, 84.6% of singletons were term births, decreasing to 35.6% in twins and only 7% in high order multiples.

**Figure 7:**
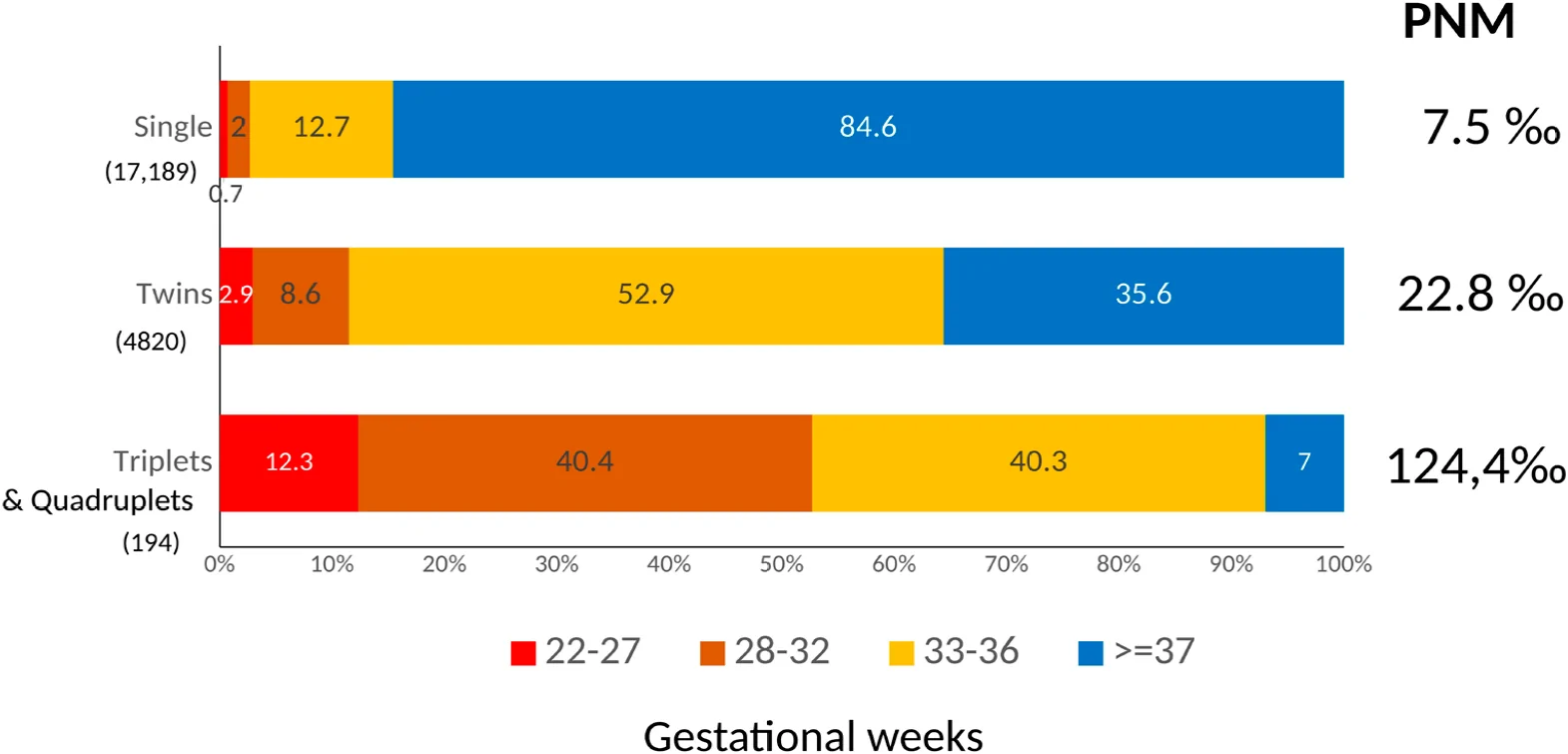
Data on 19,663 deliveries, perinatal mortality (PNM) and gestational age at birth in 20,203 births. Latin American ART Registry, 2022.

### Fertility preservation (FP)

In the last 6 years, the number of cycles for fertility preservation have increased almost 2.7 times (Zegers-Hochschild *et al*., 2025). In 2022 there were 13,227 initiated cycles (Table 1) of which 12,636 had at least one mature oocyte (MII). Following similar patterns to previous years, at the time of FP, 71.5% of women were 35 years and older, and 20.5% were ≥40 years (Supplementary Table 6). The mean (± SD) number of MII oocytes vitrified in each age group showed a significant drop as age increases. Concerning reasons for FP, in 54.8% of cases the reason was fertility postponement due to non-medical reasons, while cancer was the diagnosis in only 3.7% of cases. The rest of diagnoses (41.5%) included conditions directly or indirectly associated with risks of ovarian insufficiency.

## DISCUSSION

This is the 34^th^ report on ART procedures performed in Latin America in 2022. It includes ART outcome reported by 204 certified institutions in 16 countries (Supplementary Table 1). The majority (73.7%) of the 123,265 ART cycles reported here come from Brazil (40.6%), followed by Mexico (17.1%) and Argentina (16.2%). To have the data included in the Latin American Registry, centers need to be accredited by an independent body from a different country. Many small centers in the region are not prepared or willing to open their facilities to an external certifying body and therefore do not provide data to REDLARA. An interesting example is Brazil, where only 68 out of 186 centers registered in the Brazilian national regulatory body (ANVISA) report to REDLARA; however, the number of cycles collected from reporting centers account for 98.4% of all ART cycles officially registered in Brazil. A similar situation takes place in Argentina and Colombia. These real-world data have been collected since 2009 as the first multinational registry; and has remained as a voluntary reporting program for 35 years. It started as a summary data collection system and since 2012 evolved towards a cycle-based data collection system (DCS), with a software developed by REDLARA and providing detailed information on different treatment alternatives and their outcome. It is also an invaluable source, used for the analysis of regional trends over the years.

When examining the information presented here, it is important to consider that in contrast to national registries, in this multinational registry, results reflect the realities in 16 countries with different reproductive strategies, differences in access to treatment, limitations in the number of embryos transferred, age distribution of women.

Since its start, the agreement has been that results from specific institutions are not to be disclosed. Therefore, all results presented in this report provide data on means and medians from numerous centers with highly heterogeneous results. An example of this is described in Figure 8, showing the delivery rate after the transfer of one frozen/thawed embryo (SET-FET) in women of all ages; with and without PGT. The median DR/ET was 26% without PGT and 40% with PGT. However, the mean delivery rate in centers in the 10^th^ and 20^th^ decile fluctuated between 13.3% and 16.6% without PGT and 19.1% and 28.6% with PGT, while a similar number of centers with results located in the highest two deciles, reported a DR per SET of 39.6% and 46.3% without PGT and 49.4% and 53.7% with PGT. These are important aspects to consider when analyzing global data. As part of the software developed by REDLARA, each center can access their own data as well as the global data from their country and from Latin America. It is therefore possible for each institution to have an external quality control in relationship to its country and region. Separating results in percentiles is yet another way to evaluate the performance of each center and how they change over the years. This is also a valuable tool for the re-accreditation process which takes place every 2 to 3 years.

**Figure 8:**
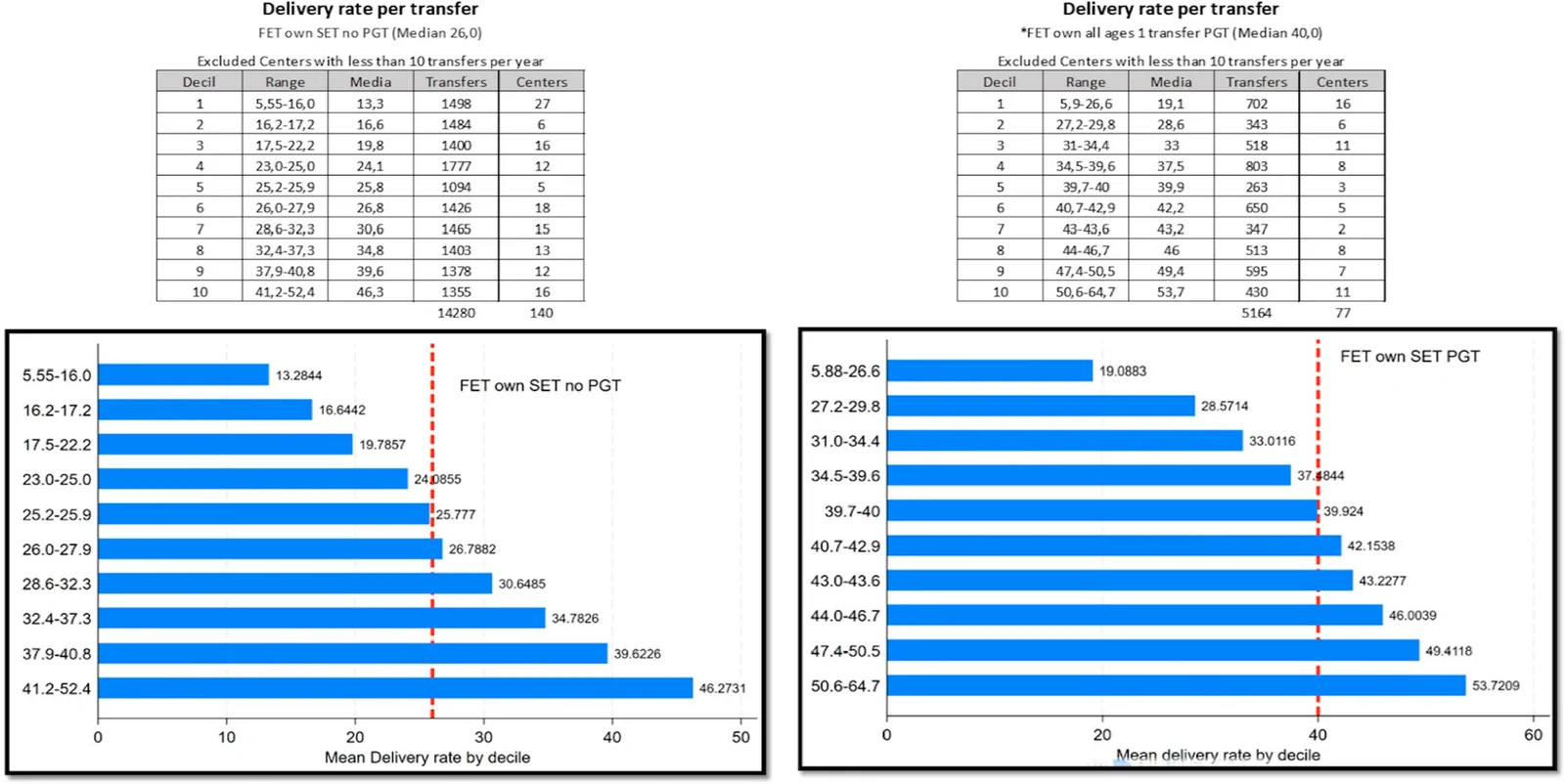
Variability of outcome reported by different ART centers expressed in deciles. Delivery rate per one embryo transferred (SET) in autologous FET with and without PGT. Latin American ART Registry 2022.

Perhaps one of the most important challenges faced by Latin American countries results from inequality in access to ART. The estimated mean utilization (number of ART cycles per million inhabitants) in Latin America is only 242.8 cycles per million, compared with 654 in the world, with great fluctuations, from 126 in Africa to over 3000 cycles in Japan, Australia, and a mean of 1342 cycles /million in Europe (Dyer *et al*., 2025). Considering only countries where most cycles (at least 75%) are reported to RLA, ART utilization fluctuates from 28.8 in Guatemala to 643.3 in Uruguay. The reason for this poor access to ART results from out-of-pocket funding as the only means to access treatment in most countries. As a result of poor access, the proportion of children born from ART is 0.33% of all births in the region; again, with huge fluctuations from 0.04% in Guatemala to 1,03% in Uruguay.

### Fresh or frozen/thawed transfer?

In Latin America, the preference for frozen-thawed transfers continues to rise over fresh embryo transfer. This is strongly influenced by a more than two-fold increase in the use of PGT in the past 5 years. In 2022, FET cycles represented 74.2% of all autologous transfers; and 87.8% of these transfers were blastocysts, compared with only 57.8% in fresh transfers. This higher proportion of blastocyst transfer in FET cycles is what explains the higher delivery rate after FET without PGT (30.1%) compared with fresh transfers (25.3%) (Tables 2 and 4). However, in a subgroup of only elective fresh blastocyst transfers (fresh blastocyst eSET), the delivery rate increased to 32.6%, significantly higher than a single FET (28.4%) (*p*<0.0001). Furthermore, the delivery rate after the fresh transfer of a single embryo generated from vitrified warmed oocytes (in OD) was 38.0%. This high delivery rate is strongly influenced by supposedly healthy donors. It also reflects improvements in oocyte vitrification which is good news for young women wishing to preserve fertility. Lastly, when comparing delivery rates after embryo transfer in a monitored natural cycle (14.2% of all FET) and after an hormonally replaced cycle (85.8% of all FET), there were no differences in the clinical pregnancy rates, while the delivery rate per transfer was in the border of significance in favor of monitored natural cycle. This difference follows similar results to those reported in a well-designed study by Jin *et al*. (2021) and confirmed in a randomize controlled trial published by Liu *et al*. (2025).

### Preimplantation genetic testing (PGT)

PGT was performed in 30.8% (13,728/44,598) of all autologous aspirations with at least one mature oocyte and 15.6% (2209/14,124) of oocyte donation cycles (Figure 1 and Supplementary Figure 4). This represents more than twofold increase of PGT in autologous cycles in only 5 years (Supplementary Figure 3). Data shows that the selective transfer of euploid embryos increased the chances of births; mainly because embryos not reaching blastocysts or being aneuploid were discarded from the denominator; as well as embryos not surviving biopsy or thawing/warming. Despite all these advantages of using transfers instead of aspirations in the denominator, the use of PGT in embryos generated by oocyte donors is not justified. In our previous publication we showed that the frequency of aneuploidy was much higher in embryos from young infertile couples than in embryos generated by oocyte donors of similar age (Zegers-Hochschild *et al*., 2025). This is confirmed in this report where PGT in embryos generated by oocyte donors did not improve the chances of birth; while PGT performed in women <35 years, improve delivery rate and decreased miscarriage rate (Table 6).

The Latin American Registry of ART acknowledges the immense effort and perseverance of more than 200 centers which year after year, voluntarily share their results to this regional registry. We wish to express our deepest gratitude to Ferring Pharma for their long term support of our registry. Furthermore, for the first time, REDLARA together with the University Diego Portales in Santiago, Chile have used this robust database to develop an educational portal for the education and empowerment of patients and to facilitate the interaction with their reproductive health care providers. This educational portal in Spanish, English and Portuguese can be accessed at: www.redlara.org

## Figures and Tables

**Supplementary Figure 1:**
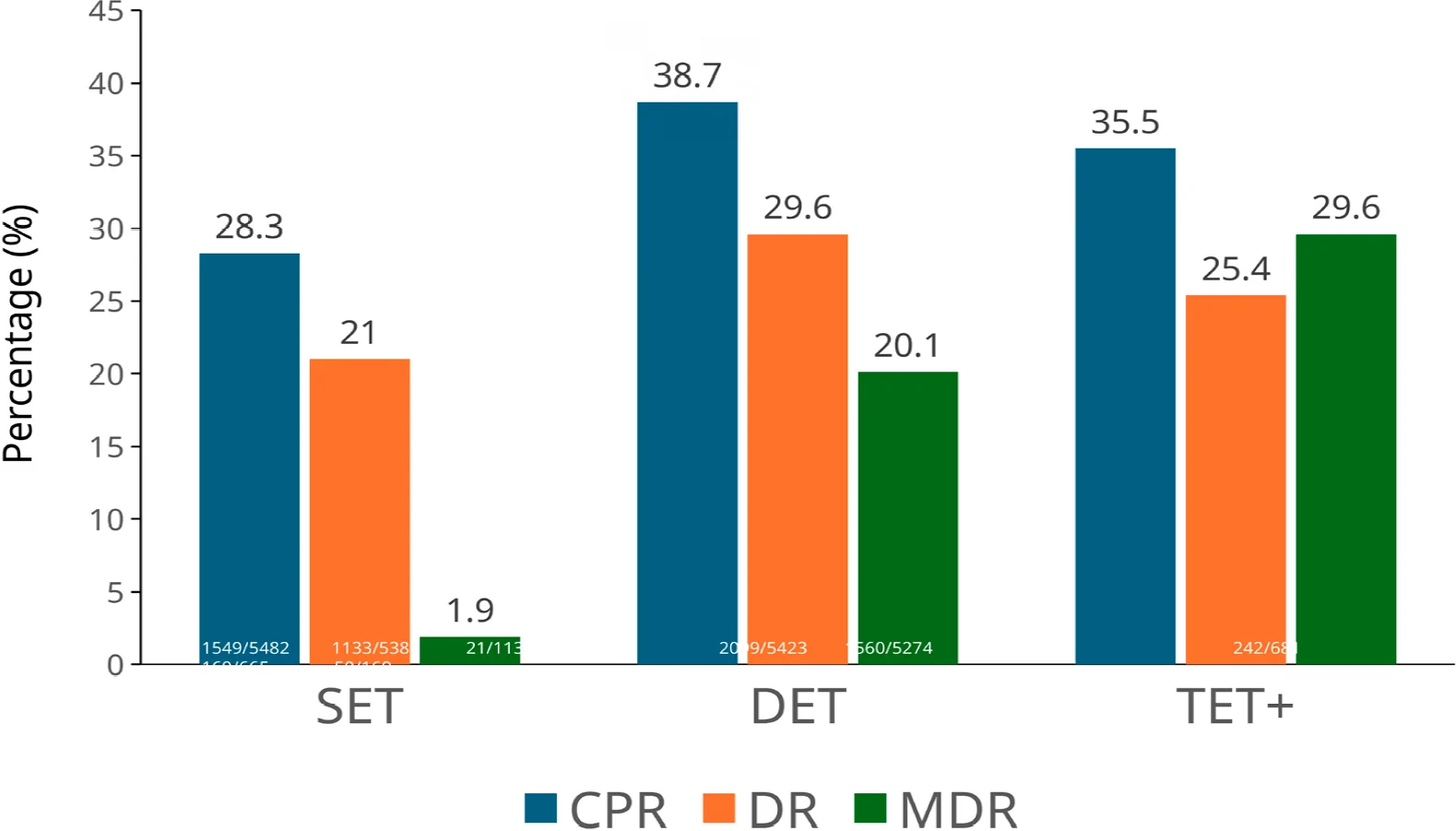
Clinical pregnancy rate (CPR), delivery rate (DR) and multiple delivery rate (MDR) per embryo transfer in autologous fresh IVF and ICSI cycles according to the number of embryos transferred in Latin American ART Registry, 2022. SET: single embryo transfer. DET: double embryo transfer: TET+: three or more embryos transferred.

**Supplementary Figure 2:**
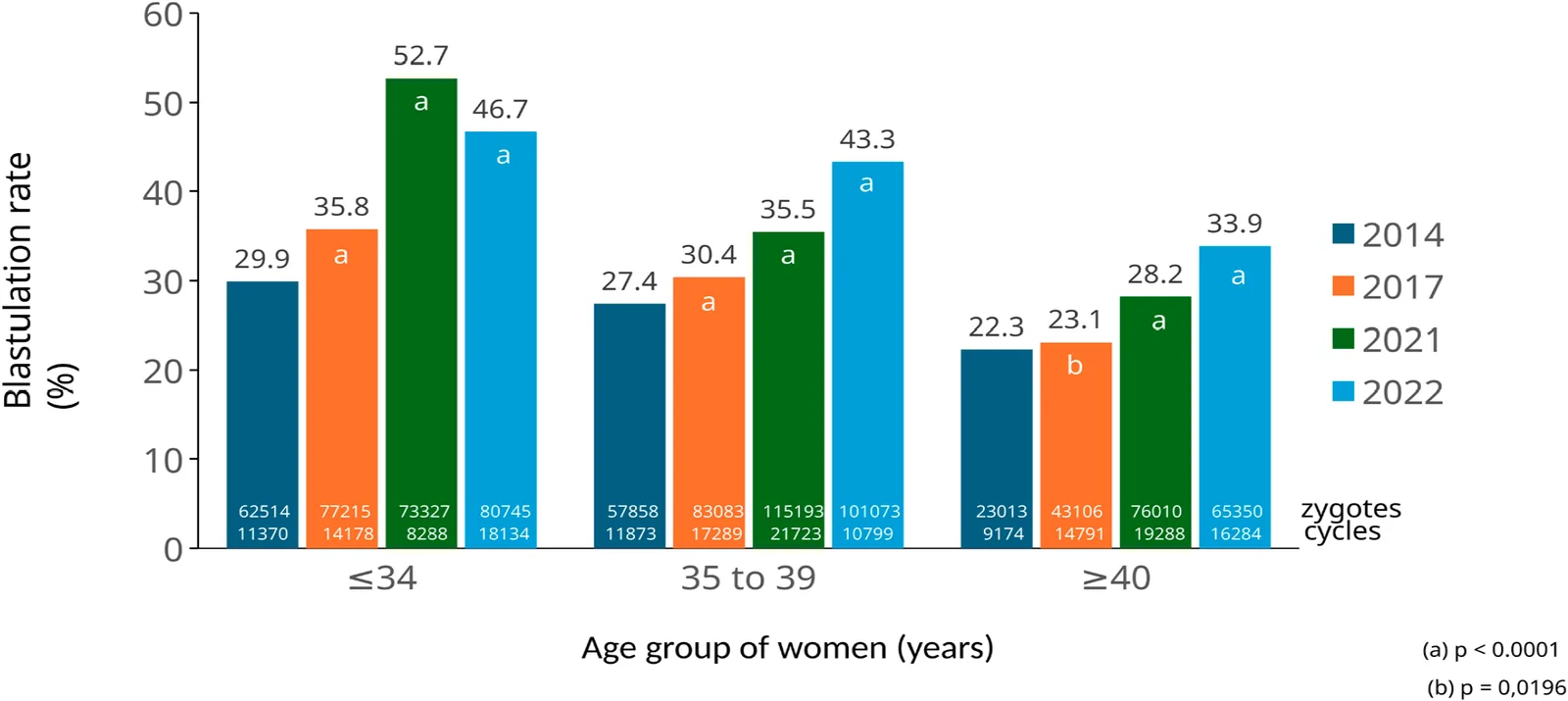
Blastulation rate (# transferred blastocysts + # vitrified blastocysts / # zygotes) according to women’s age group between 2014 and 2022. Latin American ART Registry 2014, 2017, 2021 and 2022. Letters ‘a’ and ‘b’ indicate significant differences versus 2014.

**Supplementary Figure 3:**
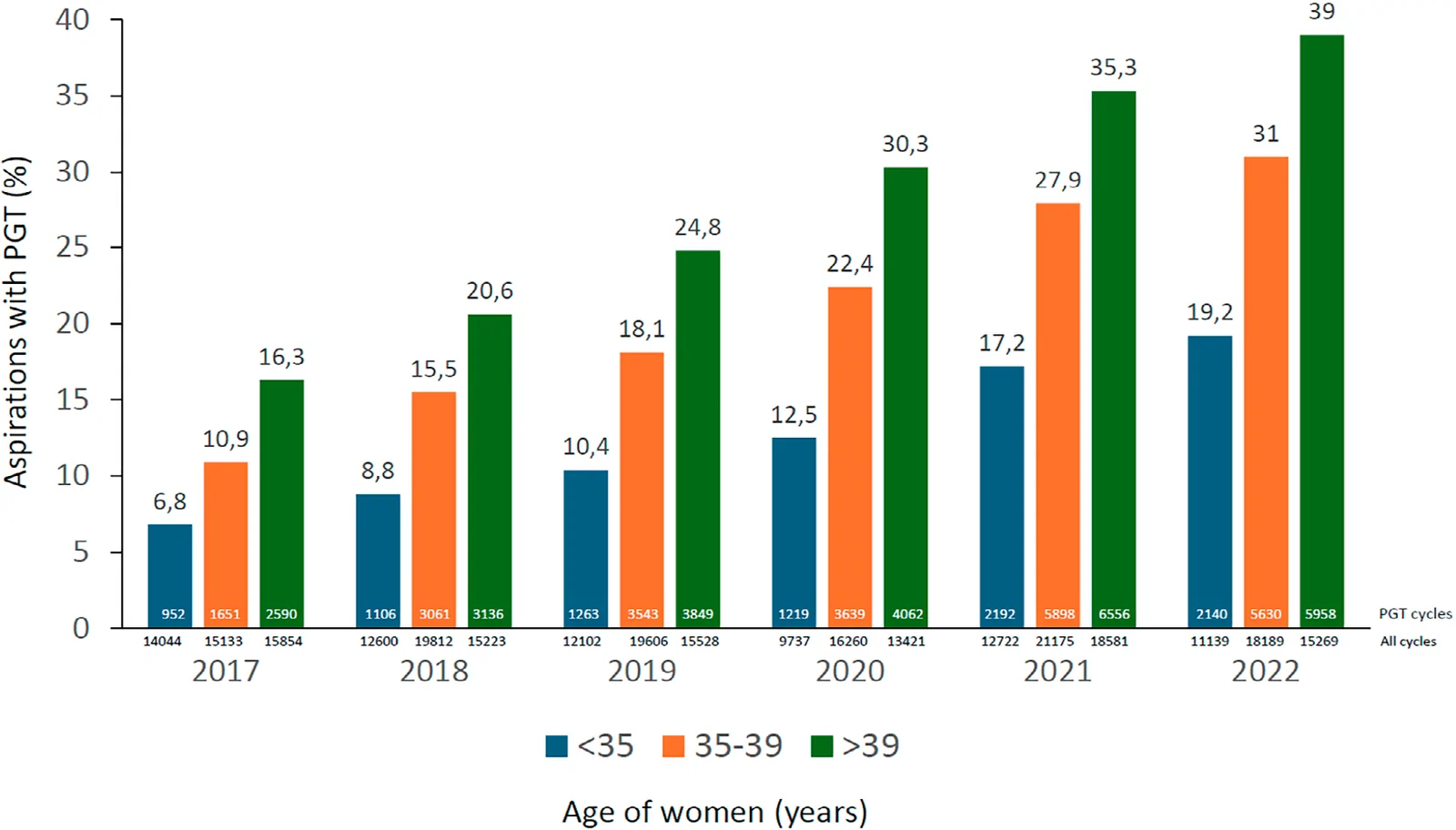
Six years trend on the use of PGT in different age groups for autologous cycles. Latin American ART Registry 2017-2022.

**Supplementary Figure 4:**
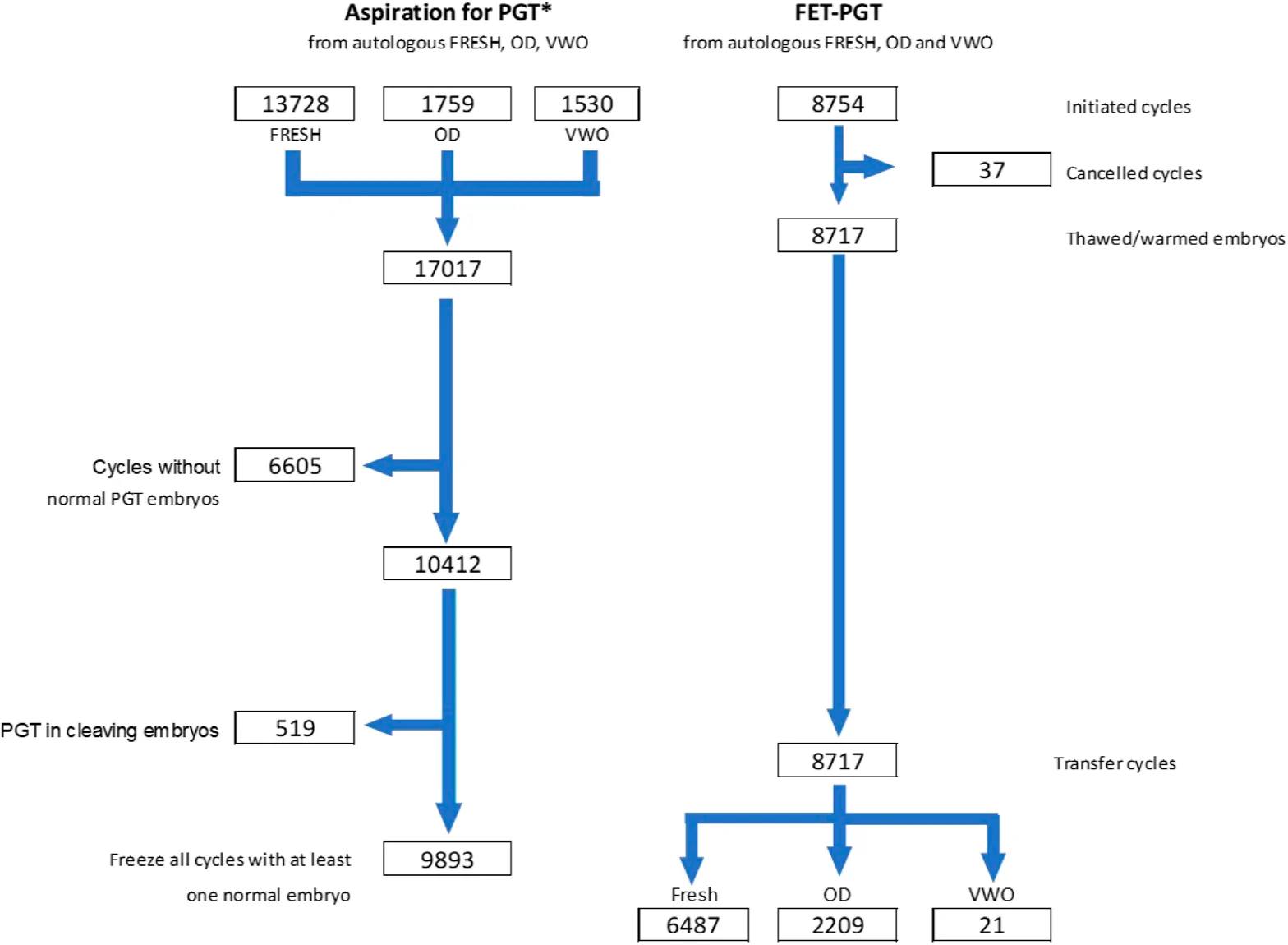
Events that affect the outcome of PGT cases. * Includes aspirations from autologous and oocyte donation (OD) cycles as well as vitrified/warmed oocytes (VWO) in Latin American ART Registry, 2022.

**Supplementary Table 2:** Clinical pregnancy rate, delivery rate and gestational order in elective and non-elective blastocyst SET and DET in Fresh autologous IVF/ICSI in 2022

Type of transfer	Embryo transfers		Clinical pregnancies		Embryo transfers^*^	Deliveries							
	Number	%	Number	%	Number	Number of deliveries	Delivery rate per embryo transfer^*^(%)	Singleton (n)	Singleton (%)	Twin (n)	Twin (%)	≥Triplets (n)	≥Triplets (%)
oSET	1646	45.4	419	25.5	1619	294	18.2	291	99.0	3	1.0	0	0
eSET	1982	54.6	837	42.2	1935	631	32.6	621	98.4	10	1.6	0	0
oDET	1526	50.2	563	36.9	1470	404	27.5	327	80.9	76	18.8	1	0.3
eDET	1515	49.8	802	52.9	1461	608	41.6	453	74.5	150	24.7	5	0.8

* Excluding cases of pregnancies with loss of follow-up (LFU).

eSET, elective single-embryo transfers; oSET, transfer of only one embryo because there are no more embryos available for transfer; eDET, elective double-embryo transfers; oDET, the transfer of only two embryos because there are no more embryos available for transfer.

**Supplementary Table 3:** Clinical pregnancy rate, delivery rate and gestational order according to the number of fresh embryos transferred in oocyte donation cycles in 2022.

Number of embryos transferred	Embryo transfers^*^		Clinical pregnancies		Embryo transfers^**^	Deliveries							
	Number	%	Number	CPR (%)	Number	Number of deliveries	Delivery rate per embryo transfer^*^(%)	Singleton (n)	Singleton (%)	Twin (n)	Twin (%)	≥Triplets (n)	≥Triplets (%)
1	1770	47.7	761	43.0	1746	590	33.8	583	98.8	7	1.2	0	0
2	1675	45.2	886	52.9	1626	717	44.1	527	73.5	189	26.4	1	0.1
≥3	262	7.1	142	54.2	245	113	46.1	81	71.7	27	23.9	5	4.4
Total	3707	100	1789	48.3	3617	1420	39.3	1191	83.9	223	15.7	6	0.4

* Excludes cases with PGT.

** Excluding cases of pregnancies with loss of follow-up (LFU).

**Supplementary Table 4:** Clinical pregnancy rate, delivery rate and gestational order according to the number of frozen embryos transferred in oocyte donation cycles in 2022.

Number of embryos transferred	Embryo transfers^*^		Clinical pregnancies		Embryo transfers^**^	Deliveries							
	Number	%	Number	CPR (%)	Number	Number of deliveries	Delivery rate per embryo transfer^*^(%)	Singleton (n)	Singleton (%)	Twin (n)	Twin (%)	≥Triplets (n)	≥Triplets (%)
1	4445	54.2	1852	41.7	4347	1417	32.6	1390	98.1	27	1.9	0	0.0
2	3287	40.0	1669	50.8	3159	1273	40.3	935	73.4	331	26.0	7	0.5
≥3	476	5.8	277	58.2	462	222	48.1	124	55.9	87	39.2	11	5.0
Total	8208	100	3798	46.3	7968	2912	36.5	2449	84.1	445	15.3	18	0.6

* Excludes cases with PGT.

** Excluding cases of pregnancies with loss of follow-up (LFU).

**Supplementary Table 5A:** Clinical pregnancy rate, delivery rate and gestational order after the transfer of fresh embryos developed from donated vitrified-warmed oocytes, 2022.

Number of embryos transferred	Embryo transfers^*^		Clinical pregnancies		Embryo transfers^**^	Deliveries							
	Number	%	Number	CPR (%)	Number	Number of deliveries	Delivery rate per embryo transfer^*^(%)	Singleton (n)	Singleton (%)	Twin (n)	Twin (%)	≥Triplets (n)	≥Triplets (%)
1	1222	58.8	604	49.4	1172	445	38.0	440	98.9	5	1.1	0	0.0
2	828	39.9	390	47.1	791	285	36.0	203	71.2	80	28.1	2	0.7
≥3	27	1.3	10	37.0	27	10	37.0	9	90.0	0	0.0	1	10.0
Total	2077	100	1004	48.3	1990	740	37.2	652	88.1	85	11.5	3	0.4

* Excludes cases with PGT.

** Excluding cases of pregnancies with loss of follow-up (LFU).

**Supplementary Table 5B:** Clinical pregnancy rate, delivery rate and gestational order after the transfer of cryopreserved embryos developed from donated vitrified-warmed oocytes, 2022.

**Cryopreserved embryos from donated VWO cycles**	Embryo transfers^*^	**Clinical** **pregnancies**		Embryo transfers^**^	**Deliveries**							
	**Number**	**Number**	**CPR (%)**	**Number**	**Number of deliveries**	Delivery rate per embryo transfer^*^(%)	**Singleton (n)**	**Singleton (%)**	**Twin** **(n)**	**Twin** **(%)**	**≥Triplets (n)**	**≥Triplets** **(%)**
Cryopreserved	1295	512	39.5	1236	375	30.3	347	92.5	28	7.5	0	0.0

* Excludes cases with PGT.

** Excluding cases of pregnancies with loss of follow-up (LFU).

**Supplementary Table 6:** Fertility preservation in women at different age groups in Latin America ART Registry, 2022.

**Age**	**n (%)**	**Mean number oocytes vitrified (SD)**	**Significance with 95% confidence Interval with respect to previous age range**
<30	1088 (8.6%)	12.06±8.19	----
30 - 34	2519 (19.9%)	9.19±6.78	*p*<0.0001 (95% CI -3.3845 to -2.3555)
35 - 37	4034 (31.9%)	8.32±6.26	*p*<0.0001 (95% CI 0.5482 to 1.1918)
38 - 39	2409 (19.1%)	6.71±5.34	*p*<0.0001 (95% CI 1.3105 to 1.9095)
40 - 42	1942 (15.4%)	5.52±4.49	*p*<0.0001 (95% CI 0.8923 to 1.4877)
>42	644 (5.1%)	4.34±3.88	*p*<0.0001 (95% CI 0.7925 to 1.5675)
Total	12,636 (100%)	7.88±4.52	----

Data exclude 591 initiated cycles with no mature oocytes.

## Notes

**Supplementary Table 1.** 204 centers in 16 countries

ARGENTINA

Servicio de Medicina Reproductiva, Instituto Gamma

Centro de Estudios en Ginecología y Reproducción (CEGYR)

Centro Integral de Ginecología, Obstetricia y Reproducción (CIGOR)

Centro de Medicina Reproductiva Bariloche , Fertility Patagonia

Centro de Estudios en Reproducción y Procedimientos de Fertilización Asistida (CRECER)

FERTILAB

Fertilis Medicina Reproductiva

Fertya

FECUNDART

Centro de Reproducción, servicio de Ginecología Hospital Italiano

Nascentis, Medicina Reproductiva

HALITUS, Instituto Médico

PREGNA, Medicina Reproductiva

Programa de asistencia reproductiva PROAR

PROCREARTE

SARESA, Salud reproductiva Salta

VITAE, Medicina Reproductiva

BOLIVIA

CENALFES

Instituto de Salud Reproductiva (ISARE)

EMBRIOVID, sede la PAZ

EMBRIOVID, sede Santa Cruz de la Sierra

BRAZIL

ANDROLAB, Clínica y Laboratorio de Reproducción Humana y Andrología

ANDROFERT, Centro de Referencia en Reproducción Masculina

FERTIVITRO, Centro de Reproducción Humana

BIOS, Centro de Medicina Reproductiva

FIV-MED

Centro de Medicina da reproduçao

VIDA, Centro de Fertilidad

Clínica FERTWAY

Nascer-Medicina Reprodutiva Ltda.

ORIGINARE, Centro de Reproducción Humana

CLINIFERT, Centro de Reproducción Humana

CONCEPTUS, Centro de Reproducción Asistida de Ceara

CONCEBER, Centro de Reproducción Humana

Clínica Origen

Clínica Pro-Gerar

Centro de Reproducción Humana CONCEPTION

Centro de Reproducción Humana MONTELEONE

Centro de reproduçao humana Wahib Hassan

Fértile Diagnósticos

CEERH, Centro especializado en Reproducción Humana

Embrios, centro de reproducción humana

EMBRYOLIFE, Instituto de Medicina Reproductiva

CENAFERT, Centro de Medicina Reproductiva

Instituto VERHUM

Clínica FERTIBABY BH

Huntington Brasilia

FECUNDA, Reproducción Humana

FELICCITA, Instituto de Fertilidad Ltda.

HUMANA, Medicina Reproductiva

FertLiv

FERTILITY, Centro de Fertilizaçao Asistida

FERTIL Reproduçao Humana

REPROFERTY

FERTICLIN, Clínica de Fertilidad Humana

LabFIV Reproduçao asistida

Genesis Instituto de reproducción humana de Cascavel PR

GENESIS, Centro de Assistencia en Reproduçao Humana

Genics, medicina reproductiva y genómica

FERTIPRAXIS

GERA, Grupo de endoscopia y Reproducción Asistida

Generar-Reproduçao humana

Clinica GERAR VIDA

Cegonha Medicina Reproductiva

Hospital de Clínicas de Riberao Preto

HUNTINGTON, Centro de Medicina Reproductiva Ibirapuera

JULES WHITE, Centro de Medicina Reproductiva

HUNTINGTON Vila Mariana

Ideia Fertil, Santo André

Ideia Fertil, Sao Paulo

IMR, Instituto de Medicina Reproductiva e Fetal

Insemine , Centro de Reproducción Humana

Centro de Reproducción Humana Santa Johana

Lab For Life Centro de Medicina Reprodutiva

Life reproducción humana

FERTILITAT, Centro de Medicina Reproductiva

Clínica Nidus

Centro de Pesquisa e Reproduçao Humana Nilo Frantz, Porto Alegre

Origen, Centro de Medidicina Reproductiva BH

Procriar, Centro de Medicina Reproductiva y diagnósticos Ltda., Blumenau

Clínica PRO-CRIAR, Medicina Reproductiva BH

Clínica PRO NASCER

Clinica ProSer

Centro de Reproducción Humana De San Jose de Rio Preto

Clinica Reproduce

Centro de fertilidad do Hospital Moinhos de vento

GENESIS, Centro de Reproducción Humana

Centro de Reprodução Humana Prof. Franco Junior

Centro de Ensino y Pesquisa en Reproducción Asistida (CEPRA)

CHILE

UMR Clínica de la Mujer Antofagasta

Centro de Estudios Reproductivos (CER)

Unidad de Medicina Reproductiva, Clínica Alemana

Unidad de Medicina Reproductiva, Clínica las Condes

Unidad de Medicina Reproductiva, Clínica de la Mujer

UMR clínica Indisa

Programa de Fertilización Asistida I.D.I.M.I.

Clínica Monteblanco

Instituto de Medicina Reproductiva Concepción S.A.

Centro de reproducción humana, Valparaíso

SG Fertility Chile

COLOMBIA

Centro FECUNDAR, Cali

Unidad de fertilidad del Coutry ltda. CONCEPTUM

Fertility Care Clinica de Fertilidad

Asociados en Fertilidad y Reproducción Humana

Clínica de la mujer

Clínica Eugin

FERTIVIDA

Clínica Machicado SAS

Centro Médico IMBANACO

Instituto de Fertilidad Humana S.A.S. (INSER Bogotá)

IN SER, Instituto Antioqueño de Reproducción (Medellín)

NOVAFEM

Procrear

Profamilia Fertilidad

Unidad de Fertilidad, Procreación Medicamente Asistida

Unión temporal IN SER eje cafetero (Pereira)

COSTA RICA

Azul Fertility expert

ECUADOR

Biogepa-Centro de Reproducción Humana

Centro Ecuatoriano de Reproducción Humana

FertyINFES

Instituto Nacional de Investigación de Fertilidad y Esterilidad (INNAIFEST)

FERTIMEDEX

CONCEBIR, Unidad de Fertilidad

Provida Nacer Reproducción humana

Unidad fr Fertilidad Drs. Valdivieso

EL SALVADOR

Latid Fertility Center

GUATEMALA

Centro de Reproducción Humana S.A. (CER)

Centro Clínico Gestar

MEXICO

Biofertility Center

Centro de Diagnóstico Ginecológico

Clínica CERH

Dr. Cigüeña

URA, Unidad de reproducción asistida de Hospital CIMA Hermosillo

Centro de Cirugía Reproductiva y Ginecología, Unidad de Fertilización In Vitro (REPROGYN)

Instituto de Innovación Tecnológica y Medicina Reproductiva CITMER (Ciudad de México)

Centro de Innovación tecnológica y medicina Reproductiva (Monterrey)

Citmer-Centro de innovación tecnológica y medicina reproductiva Puebla

Instituto para el estudio de la Concepción Humana IECH

Centro de Reproducción Asistida del Hospital Español (HISPAREP)

Centro de Reproducción Asistida de Saltillo

CREASIS SC

Centro Universitario de Medicina Reproductiva

Eligen Fertility Center

Fertilidad Integral

Fertility Center Cancún

Fertilita Medicina Reproductiva, Laboratorio in vitro

Centro de Medicina reproductiva Filius

Genesis Centro de Fertilidad (Culiacán)

Ginecología y Reproducción Asistida GYRA

Unidad de Medicina Reproductiva del Hospital Ángeles del Pedregal

IECH de Baja California

Instituto Mexicano de Alta Tecnología Reproductiva S.C. (INMATER)

Concibo

Instituto Médico de la mujer (RED CREA)

Instituto VIDA Guadalajara-Instituto de Ciencias en Reproducción Humana

Instituto de Ciencias en Reproducción Humana, VIDA sede Matamoros

Centro especializado para la atención de la mujer (CEPAM)

INGENES DF

INGENES Guadalajara

INICIAR

Ingenes Monterrey

Instituto de Ciencias en Reproducción Humana (VIDA), sede León

MasFertil

Instituto de ciencias en reproducción humana del Sureste (Vida Mérida)

Clínica Nascere

Centro Origen, Mérida

Plenus, Reproducción Asistida

PROGEN

Clínica de Infertilidad y reproducción asistida de Toluca SA de CV

Centro especializado en esterilidad y reproducción humana SC

UNILIVE

ViaFERT

Instituto de Ciencias en reproducción humana VIDA, ciudad de México.

Centro CARE

Vida, Instituto de Reproducción Humana del Noroeste, Tijuana

PANAMA

IVI Panamá S.A.

Panamá Fertility Clinic

Instituto de salud femenina

FERTICLINIC

PARAGUAY

Neolife, Medicina y cirugía reproductiva

PERU

UMR Clinica Delgado Auna

Clínica CEFRA, Centro de Fertilidad y Reproducción Asistida

CEFERGIN

CERAS

Centro de Fertilidad y Ginecología del Sur (CFGS)

Clínica de fertilidad del norte, Clinifer de Chiclayo

EmbryoFertility Biomedicicna Reproductiva

FERTILITA

FERTILAB

Centro de Fertilidad Germinar

Inmater, Clinica de fertilidad y reproducción asistida

Instituto de Reproducción de la Clínica Ricardo Palma

Clínica Miraflores, Instituto de Ginecología y Fertilidad

Nacer, Centro de Reproducción Humana de Lima

NiuVida

Grupo Pranor San Isidro, Clínica CONCEBIR

Grupo Pranor, Instituto de Ginecología y Reproducción Monterrico

REPUBLICA DOMINICANA

Instituto de reproducción y ginecología del Cibao - IREGCI

Programa de fertilización asistida y medicina perinatal - PROFERT

URUGUAY

Centro de Esterilidad Montevideo (CEM)

Centro de Reproducción Humana del Interior

VENEZUELA

FERTILAB

Avila FIV

Instituto Venezolano de fertilidad.
